# A rare case of symptomatic vascular ring

**DOI:** 10.1016/j.radcr.2022.11.040

**Published:** 2023-01-05

**Authors:** Marcello Chiocchi, Mario Laudazi, Paola Leomanni, Lucia Giudice, Matteo Madonna, Francesco Garaci, Roberto Floris

**Affiliations:** Diagnostic Imaging Unit, Department of Biomedicine and Prevention, University of Rome Tor Vergata, Viale Oxford 81, Rome, 00133, Italy

**Keywords:** Congenital vascular anomalies, Double aortic arch, Vascular ring, Incidental finding, Subclavian steal syndrome

## Abstract

In this paper, we describe a rare case of double aortic arch with dominant right arch with focal narrowing of the distal left arch and descendent aorta's dilatation, associated with pulmonary embolism and left subclavian steal syndrome, found in a 59-year-old woman with a history of dysphagia, chest discomfort, and left arm claudication. Diagnosis of this condition was made with a sub-optimal pulmonary CT-angiography with a combination of characteristic features of double aortic arch and vascular rings. Being aware of these conditions is crucial to avoid misclassification and surgical and endovascular complications.

## Introduction

Congenital variants of the aortic arch are embryologic developmental abnormalities involving the epiaortic vessels and may be associated with vascular ring, congenital cardiac abnormalities, and chromosomal abnormalities. These are conditions generally found in pediatric age [1]; however, they can also be discovered in asymptomatic adults, or associated with symptoms such as dysphagia, respiratory distress, and other disorders that alter the prognosis and surgical management of the patient [2]. This makes a thorough understanding of the various possible malformations and their differential diagnosis essential.

In this case report, we describe a rare case of symptomatic vascular ring formed by asymmetrical double aortic arch (DAA) with a focal narrowing of the distal portion of the left aortic arch associated with the presence of an aortic sacciform dilatation resembling an aortic diverticulum, subclavian steal syndrome, and pulmonary embolism in an adult patient.

## Case report

A 58-year-old female patient arrived in the emergency department following onset of acute chest pain radiating to the left arm and dyspnea. The patient presented with clinical history of hypertension, lung carcinoma localized to the right upper lobe with lymph node metastasis, and secondary involvement of the left lung. A high-flow pelvic varicocele and a large pelvic arteriovenous malformation were treated with endovascular techniques [3,4]. The patient also reported history of dysphagia and pain during swallowing, functional limitation, and paresthesia in the left upper limb.

On admission to the emergency department, on suspicion of acute myocardial ischemia, the patient performed laboratory examinations that showed no increase in markers of myocardial necrosis; however, an increase in D-dimer value of 1153 ng/mL (maximum reference value: 500 ng/mL) was shown.

In relation to the patient's symptoms and laboratory findings, on suspicion of pulmonary thromboembolism, a pulmonary CT-angiography was performed by administering 70 mL of intravenous Iomeprol (Iomeron 350 mgI/mL) at high flowrate (4 mL/sec).

The investigation revealed an occlusive defect of a segmental arterial branch of the posterior segment of the right lower lobe, which confirmed the clinical suspicion of pulmonary embolism. However, the study also revealed the presence of an anatomical abnormality of the aortic arch and its main epiaortic branches.

A right aortic arch of regular size originated from the sinus-tubular junction (ascending aorta diameter 33 mm). The descending aorta ran in the right paramedian line in its middles third and then lateralized to the left passing regularly through the aortic diaphragmatic hiatus.

A smaller left arch (mean diameter of 9 mm) was also observed, which appeared stenotic and hypoplasic in its posterior segment at its junction with a sacciform dilatation of the left lateral wall of the descending aorta (diameter 45 mm), resembling an aortic diverticulum in communication with the arch.

**Fig. 1:**
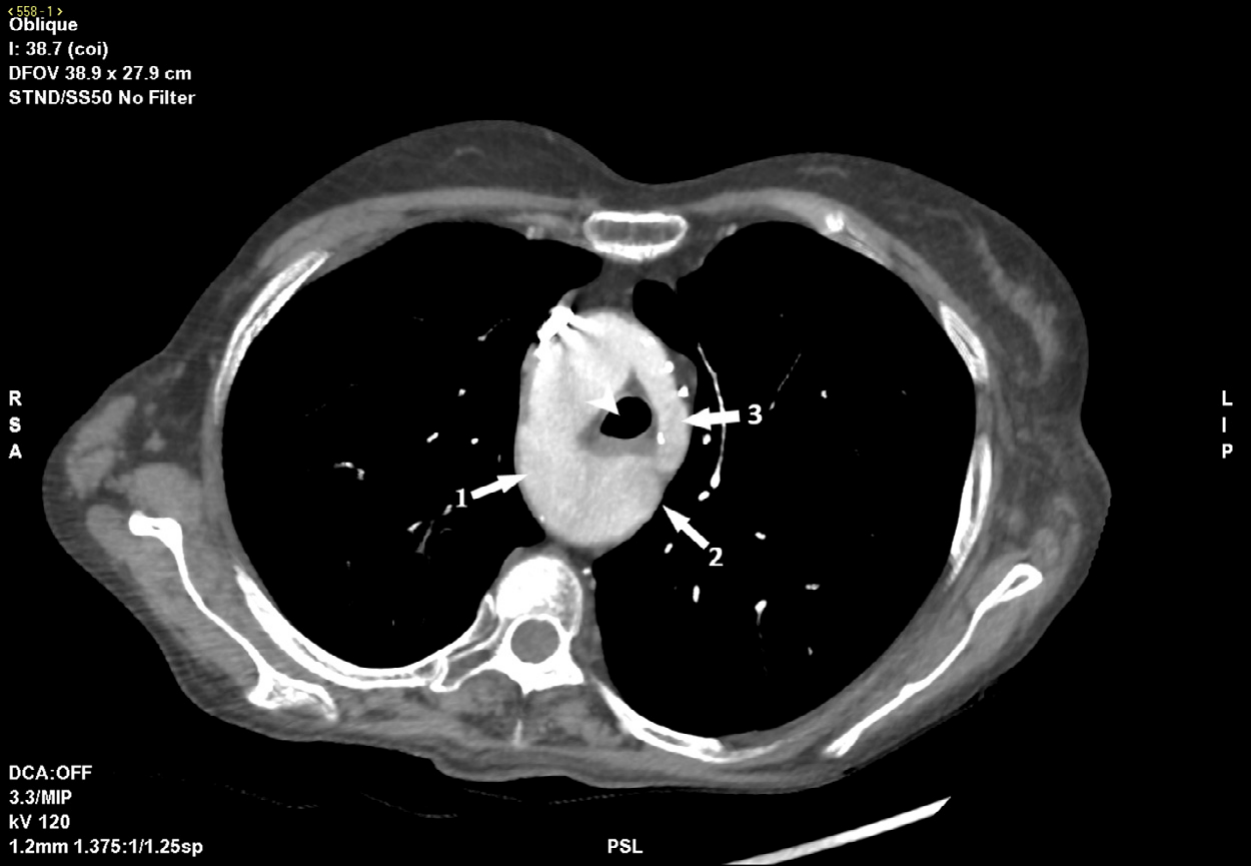
Oblique-axial MIP view of angiographic-CT-scan, showing evidence of a vascular ring surrounding the middle tract of the trachea and esophagus (white arrowhead), determined by the right aortic arch (white arrow nº1), the aortic diverticulum (white arrow nº2) and the residual left arch (white arrow nº3).

The esophagus appeared compressed due to the presence of a vascular ring formed by the right-posterior aortic arch, the dilatation of the descending aorta, and the distal portion of the left arch, in the absence of significant tracheal stenosis (Fig. 1).

The right subclavian artery and common carotid artery originated independently from the right aortic arch; the left common carotid artery and left subclavian artery also originated independently from the left vascular arch, respectively as the first and second vessel from the homolateral arch, configuring an aberrant retroesophageal origin of left subclavian artery.

**Fig. 2:**
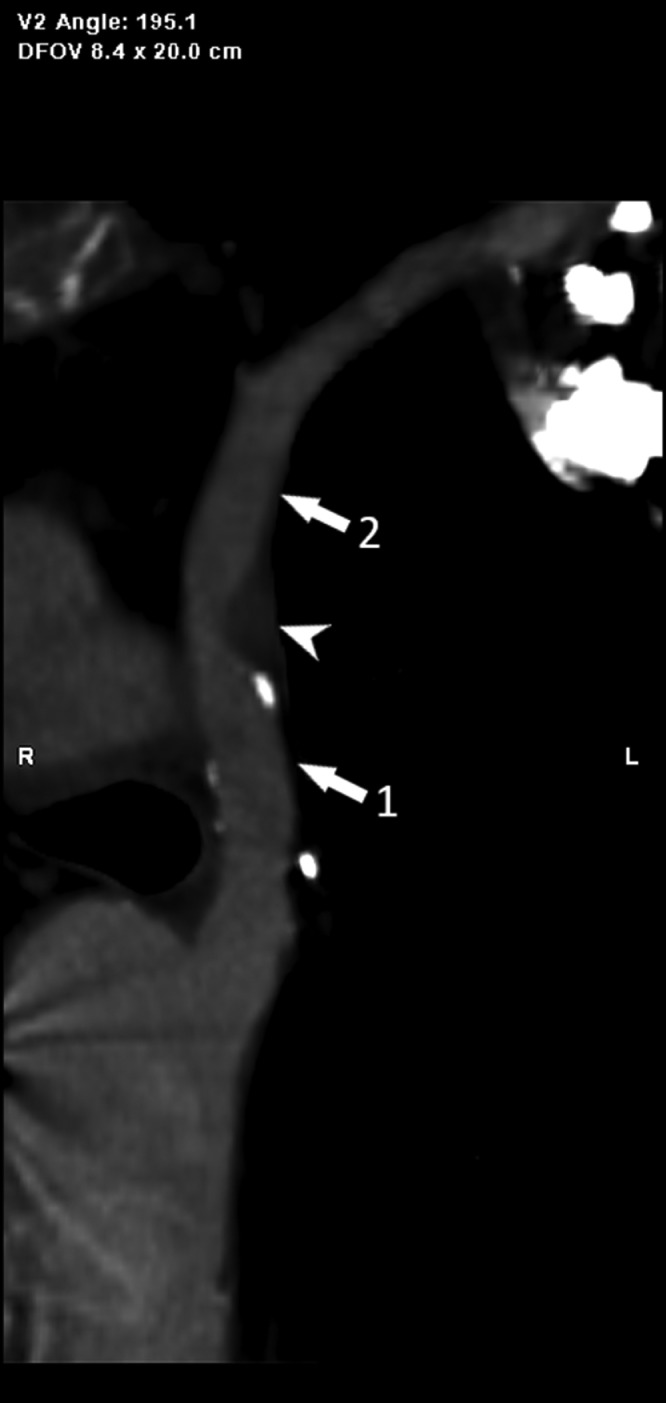
Post-processing curved reconstruction of the left aortic arch (white arrow nº1) and the left subclavian artery (white arrow nº2) showing the presence of a mixed plaque at its origin tract (white arrowhead), responsible for a significant stenosis.

**Fig. 3:**
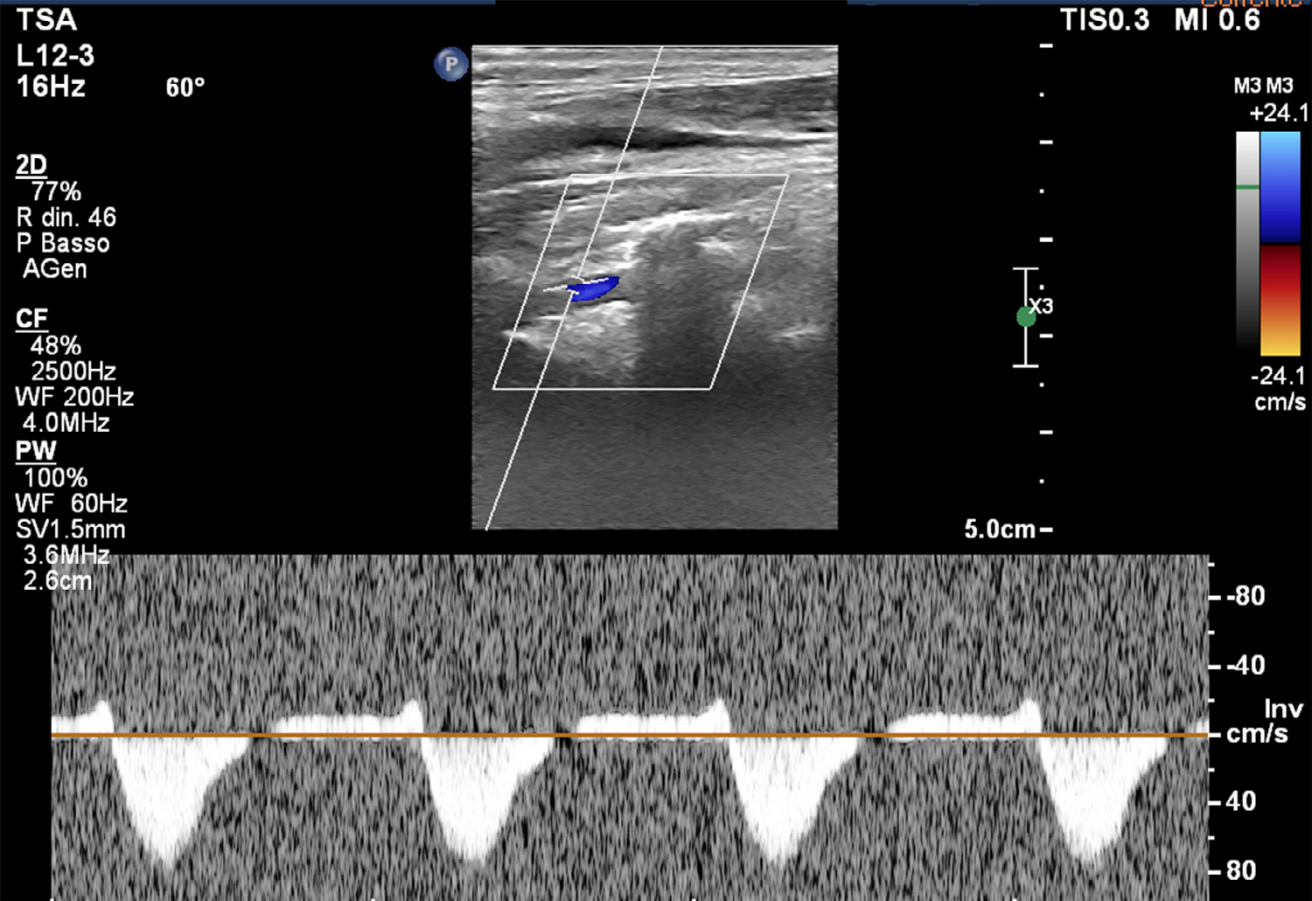
Flow-velocimetric evaluation of the left vertebral artery showing significant flow reversal on echo-color-Doppler examination.

A concentric atherosclerotic plaque of mixed composition was also observed at the origin of the left subclavian artery, determining a focal stenosis at that level of about 80% (Fig. 2). Because of the latter finding and persistent algic symptoms and fatigability at the level of the patient's left upper limb, an echo-color-Doppler examination was performed for the study of the epiaortic vessels on the left and for the static and dynamic study of the arterial vessels of the ipsilateral upper limb. The test revealed altered flow in the left vertebral and subclavian arteries and a velocimetric tracing suggestive of subclavian steal syndrome (increased PSV in the subclavian artery and reversed flow of the vertebral artery), a picture that became even clearer during the dynamic study phase (Fig. 3).

Patient underwent multidisciplinary evaluation and was treated conservatively for her acute pulmonary thromboembolic pathology. An angiographic CT study and elective surgical videat were scheduled for proper characterization and management of her vascular abnormalities; however, she was subsequently lost to follow-up.

## Discussion

DAA is an entity that results from the persistence of both the left and right aortic arches. A DAA is defined when there are 2 transverse aortic arches running over the trachea and both bronchi. In this situation, the common carotid artery and subclavian artery originate from each ipsilateral arch with sometimes a partial predominance of one of the 2 arches, which is the dominant one [5]. The ductus arteriosus usually persists as an arterial ligament from the involution of the sixth arch and connects the descending aorta with the left pulmonary artery [6]. It is usually difficult to visualize in CT studies, but we believe that in our case the ligamentum arteriosus connected the aortic diverticulum to the left pulmonary artery. In adults, it usually presents asymptomatically; however, it can present with esophageal compression symptoms such as dysphagia and breathing difficulties due to tracheal stenosis [5].

The aortic arch and its branch vessels can be thoroughly evaluated in relation to the surrounding structures using non-invasive imaging techniques like CT-angiography and MR-angiography. For a precise diagnosis and to direct treatment, it is crucial to be familiar with the range of variations, abnormalities, and malformations and their imaging manifestations. Studying these abnormalities by means of a pulmonary CT-angiographic study is still possible through accurate knowledge of the major abnormalities. In fact, ours is not the first case of anomalies of the arch and epiaortic vessels incidentally discovered through pulmonary CT-angiography in [7,8] thus making it necessary to be aware and able to classify them correctly.

Malformations of the aortic arch system can be explained by persistence of segments of the embryologic aortic arches that normally regress or disappearance of segments that normally remain [9] based on the model proposed by Edwards in 1974 [10].

The right arch is typically higher and wider than the left arch when there is a persistent DAA. The descending aorta is typically opposite the dominant arch, with the most common configuration being a larger right arch, left-sided descending aorta, and left-sided ligamentum arteriosum. In this situation, an aortic diverticulum with associated ductus arteriosus or ligamentum arteriosum is usually present, typically on the side of the atretic segment (usually the left) [6].

Focal narrowing with stenosis of the distal left arch connecting the origin of the left subclavian artery to the descendant aortic diverticulum makes the differential diagnosis between a DAA, an incomplete DAA, and a right-sided aortic arch with mirror-branching very difficult. Patency of the distal left arch is important for distinguishing a complete DAA from an incomplete DAA, where this segment is atretic, with the persistence of a fibrous cord that is normally not distinguishable in CT imaging. Instead, total involution of this segment configures a situation of right-sided aortic arch with mirror-branching, which is rarely associated with presence of a vascular ring [11,12].

**Fig. 4:**
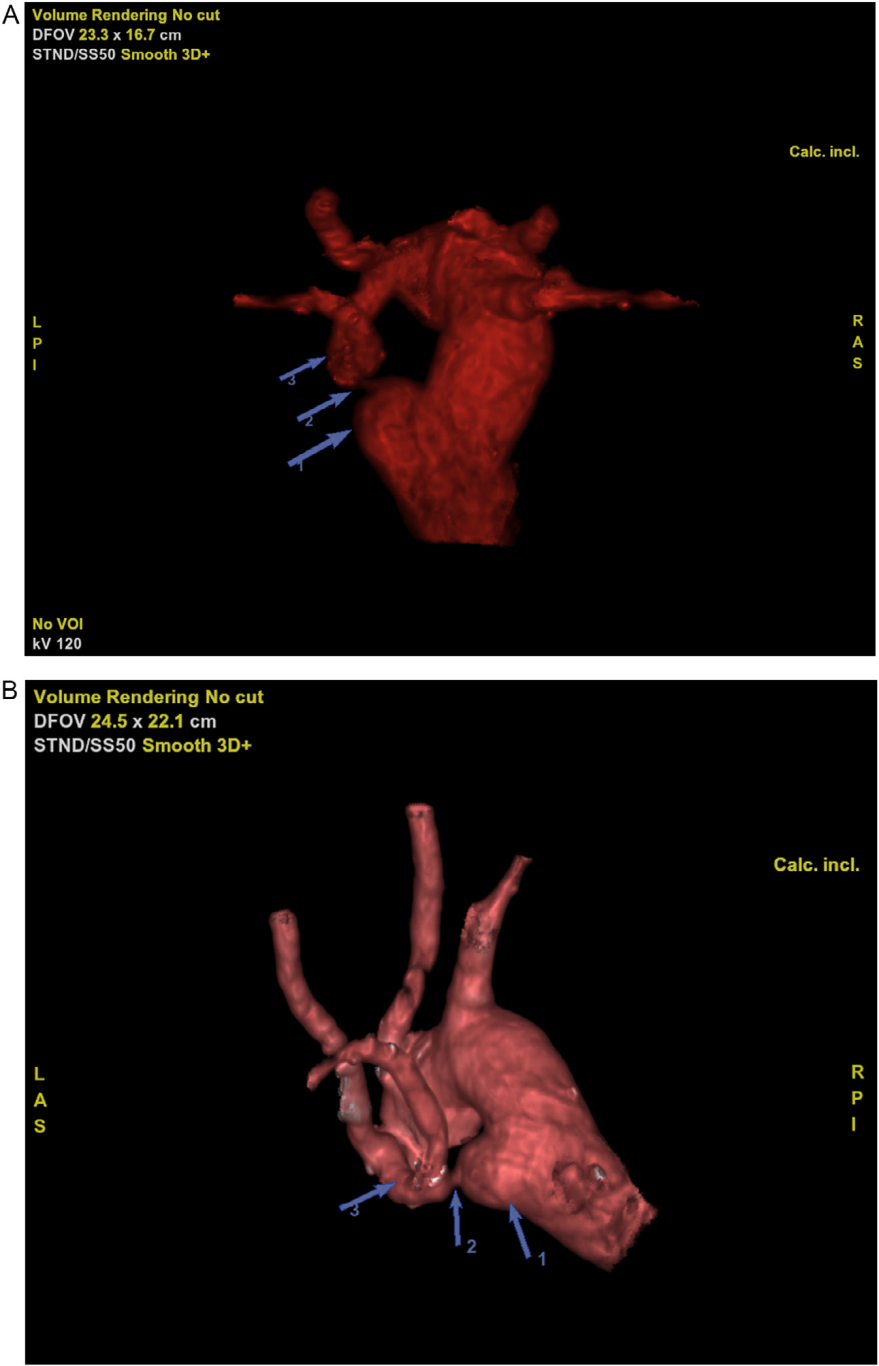
A 3D-reconstruted volume rendering of the double aortic arch with emphasis on the diverticular dilatation of the anterior wall of the thoracic aorta (1), the focal narrowing that enabled the vascular ring's genesis (2) and the residual left arch (3). A - Cranial posterior view showing the vascular ring. B - Oblique left lateral view that highlights the focal narrowing.

A DAA has common carotid and subclavian arteries originating independently from each arch, and therefore there is a symmetric appearance of the branch vessels on a transverse section just above the level of the arches (the 4-vessel or 4-artery sign). In contrast, a left brachiocephalic artery arises from a right arch with mirror image, and therefore asymmetry of the branch vessels at a transverse level just above the arch is expected [9,13] (Fig. 4A).

As imaging shows, the origin of the left common carotid artery was anterior and the origin of the left subclavian artery was especially posterior, immediately before the focal narrowing of the left aortic arch, and the intermediate part of the left aortic arch resembles a horizontal course, making it difficult to distinguish if there is an independent distal origin of the subclavian artery from a residual left arch or a short brachiocephalic artery with a long horizontalized tract of a left subclavian artery communicating with the aortic diverticulum [14] (Fig. 4B).

This distinction was made possible by the combined finding of additional features of DAA, such as the presence of symmetry of the epiaortic vessels at a more cranial level (4-artery sign), a more lateral and posterior position of the hypothetical left aortic arch, and the presence of the aortic dilatation, which are more typical in the case of an incomplete DAA or DAA [6,14].

Knowledge of the characteristic imaging findings of DAA with distal left arch narrowing is important, as this entity can still form a complete vascular ring (unlike a right aortic arch with mirror-image branching). In our case, the presence of this vascular ring likely resulted in dysphagic symptoms, as the middle tract of the esophagus was found to be between the right aortic arch and its aortic diverticulum posteriorly, the trachea anteriorly, and the distal part of the left aortic arch laterally. In addition, the presence of an aortic diverticulum may be a cause of increased risk for aortic dissection and a reason to be treated surgically [14].

In addition, moreover, the left subclavian artery had a mixed-composition plaque immediately after its origin, resulting in its stenosis of about 80%, a likely cause of claudication of the left upper limb, later confirmed by echo-color-Doppler examination that showed the inversion of the of the vertebral artery flux.

It is possible that the focal narrowing of the left arch near the origin of the left subclavian artery and its particularly aberrant course, generated blood-flow turbulency which, we infer, favored the developing of the plaque [15]. Rare cases of congenital subclavian steal have been described in literature, and some of them were associated to a presence of an anomalous right-sided aortic arch [16].

In conclusion, familiarity with aortic arch variants and epiaortic vessel abnormalities is essential to establish a correct classification and diagnosis to guide proper management of these conditions.

## Patient consent

Informed consent for publication was obtained from the patient.
